# MSCA-Net: A Multi-Scale Depthwise Attention Network for Multi-Class Intrusion Detection in Internet of Medical Things

**DOI:** 10.3390/s26134036

**Published:** 2026-06-25

**Authors:** Esra Söğüt, Mazhar Kayaoğlu, Onur Polat

**Affiliations:** 1 Department of Computer Engineering, Faculty of Technology, Gazi University, Ankara 06560, Türkiye; esrasogut@gazi.edu.tr; 2 Department of Informatics, Bingöl University, Bingöl 12000, Türkiye; mkayaoglu@bingol.edu.tr; 3 Department of Computer Engineering, Bingöl University, Bingöl 12000, Türkiye

**Keywords:** Internet of Medical Things, cybersecurity in healthcare, multi-scale learning, intrusion detection systems, imbalanced data classification, edge-based intrusion detection

## Abstract

The Internet of Medical Things (IoMT) enables real-time monitoring and decision support systems in healthcare. However, due to their heterogeneous structure, limited resources, and high criticality, IoMT networks are vulnerable to cyberattacks. This situation increases the need for low-latency, high-accuracy, and generalizable attack detection systems. In this experimental study, the Multi-Scale Depthwise Channel Attention Network (MSCA-Net) model is proposed for multi-class attack detection in IoMT environments. The model consists of three core components: multi-scale depthwise separable convolutions to capture traffic patterns across different time scales, a squeeze-and-excitation-based channel attention mechanism that adaptively weights discriminative features, and a lightweight unidirectional LSTM layer that models temporal dependencies. This architecture enables effective representation learning with low parameter costs. The proposed model was evaluated on the WUSTL-EHMS-2020 and CICIoMT2024 datasets. On the CICIoMT2024 dataset, it achieved 99.75% accuracy and a weighted F1 score of 99.77% in a 6-class scenario. It has also demonstrated competitive results in 19-class fine-grained classification. Experimental comparisons show that MSCA-Net offers a better performance-to-cost trade-off compared to nine different baseline models. Furthermore, it demonstrates a speed advantage of up to two times in inference time. The results obtained at the conclusion of the experimental study demonstrate that the proposed approach effectively addresses the challenges of multi-scale feature extraction, class imbalance, and computational efficiency. Furthermore, the model appears to offer a viable solution for real-time attack detection in IoMT environments.

## 1. Introduction

### 1.1. Background and Motivation

**Figure 1:**
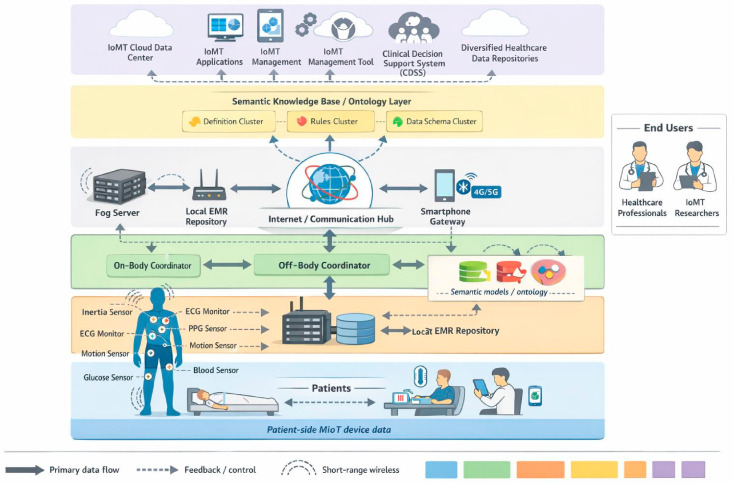
General architecture of IoMT-based healthcare systems.

Recent rapid advancements in Internet of Things (IoT) technologies have accelerated the digital transformation of healthcare, leading to the emergence of the concept of the Internet of Medical Things (IoMT). Thanks to the network-based integration of wearable sensors, implantable devices, patient monitoring systems, and various therapeutic tools, a fundamental transformation in healthcare delivery has begun with the IoMT. This framework has significantly enhanced the effectiveness and operational efficiency of healthcare services by enabling critical applications such as remote patient monitoring, continuous health tracking, and advanced diagnostic mechanisms. Additionally, IoMT systems improve clinical decision-making processes through real-time data analysis and enable remote healthcare management [1]. As shown in Figure 1, the IoMT ecosystem consists of multiple interconnected layers, including sensors, edge/fog nodes, cloud platforms, and healthcare applications.

However, the rapid proliferation of IoMT systems has also brought about serious cybersecurity risks. A review of the literature and recent incidents reveals that, particularly during the pandemic, cyberattacks in the healthcare sector have increased significantly, with IoMT devices becoming one of the primary targets of these attacks.

The highly sensitive nature of data in IoMT environments makes security breaches far more critical than in traditional IT systems. This is due to their direct impact on patient safety and human life. Data breaches, device manipulation, and service disruptions not only threaten data privacy but can also lead to system failures, resulting in interruptions to critical healthcare services [2].

One of the key factors complicating the security of IoMT systems is their highly heterogeneous and multi-protocol architecture. The communication between devices from different manufacturers via various protocols such as TCP/IP, MQTT, Bluetooth, and others brings with it differing data structures, traffic behaviors, and security vulnerabilities [3]. This situation leads to the generation of large-scale and complex network data, making it particularly difficult to detect cyber threats. Additionally, the limited processing power, memory, and energy capacity of IoMT devices restrict the implementation of traditional security mechanisms, making these systems more vulnerable to cyberattacks [4].

In this context, intrusion detection systems (IDSs) play a critical role in detecting and preventing cyber threats in IoMT environments. IDSs continuously monitor network traffic to detect abnormal behavior and provide an early warning mechanism against potential attacks [5]. In particular, machine learning and deep learning-based IDS approaches offer significant advantages due to their ability to analyze patterns in complex and high-dimensional network data. However, a significant problem stands out when reviewing current studies. Many machine learning and deep learning-based approaches fail to adequately represent the multidimensional and multi-source nature of network traffic, which can limit model performance [6].

In particular, the “Data Distribution Shift” problem demonstrates that structural differences between IoT and IoMT environments directly impact IDS performance. A review of the literature reveals that experimental studies show models trained on general IoT datasets experience significant performance drops when tested in IoMT environments. This situation clearly highlights the necessity of IoMT-specific datasets and domain-specific modeling approaches [7].

Furthermore, the real-time data flow, low-latency requirements, and high criticality of IoMT environments make the adequacy of existing IDS solutions even more questionable. Issues such as high false alarm rates, limited explainability, and a lack of adaptability to new attack types reduce the reliability of these systems in clinical settings [8]. However, a review of the literature reveals that recent studies suggest deep learning models based on multi-scale feature extraction and attention mechanisms may contribute to a more effective representation of complex IoMT traffic structures.

In conclusion, ensuring security in IoMT ecosystems is not merely a technical requirement but a critical necessity directly linked to patient safety. Therefore, the development of next-generation IDS solutions that account for the multi-protocol and heterogeneous nature of IoMT environments and possess high accuracy, low latency, and strong generalization capabilities is of great importance. In particular, advanced deep learning architectures, domain-specific datasets, and effective data representation techniques are emerging as key determinants in the construction of future secure and sustainable healthcare systems.

### 1.2. Problem Definition

Cyberattack detection in IoMT-based healthcare systems is a complex and multifaceted problem. This stems from the heterogeneous architecture, high-volume data flows, and critical requirements related to patient safety [9,10]. The diverse devices, communication protocols, and cloud integrations within the IoMT architecture expand the attack surface. This situation increases the diversity of threats while making the detection process more difficult. Therefore, attack detection must be approached differently from the classical anomaly detection methods used in information systems [11]. The detection of cyberattacks targeting IoMT-based healthcare systems should be treated as a learning problem. This problem requires the generation of reliable decisions based on multi-source, real-time data. In IoMT environments, attack detection presents itself as a multi-class problem due to the architecture and heterogeneous nature of IoMT systems [12,13]. However, many studies in the literature address the problem as a binary classification, failing to adequately reflect the true diversity of attacks. Additionally, the diversity of attack types and their constantly evolving nature limit the effectiveness of traditional methods [14].

Another significant challenge is data imbalance. The underrepresentation of rare but critical attacks prevents models from adequately learning these threats, leading to low sensitivity. Additionally, the lack of clear boundaries between normal and abnormal behavior further complicates anomaly detection.

The heterogeneous and high-dimensional nature of IoMT data complicates feature extraction and representation learning [15]. Furthermore, the large volume of data increases computational costs, while real-time requirements necessitate the design of low-latency and efficient systems. Otherwise, patient safety could be directly compromised [14,16].

Finally, data distribution shifts and varying system configurations limit the generalization ability of models. When all these challenges are considered together, it becomes evident that current IDS approaches are insufficient. Therefore, there is a clear need for next-generation solutions tailored to IoMT environments that are low-latency, high-accuracy, and generalizable.

### 1.3. Limitations of Existing Approaches

Although deep learning-based IDS methods achieve high accuracy rates, they face significant limitations, such as class imbalance, variations in data distribution, and high computational costs, which restrict their applicability in real-time IoMT environments. A review of the literature on machine learning and deep learning-based attack detection in IoMT systems reveals that existing approaches fail to fully address the unique dynamics and constraints of the IoMT ecosystem. Consequently, these approaches remain insufficient to meet the specific requirements of real-time, heterogeneous, and resource-constrained IoMT environments [17]. These limitations can be broadly categorized into the following four key groups:

- ***Limitations of Single-Scale Models:*** Most studies in the literature analyze network traffic over a fixed time window. These studies typically use standard Convolutional Neural Networks (CNNs) or Recurrent Neural Network (RNN) architectures based on a single feature scale [18]. However, IoMT traffic exhibits a multi-scale structure, ranging from sensor-based instantaneous changes to long-term protocol interactions [5]. Single-scale models are insufficient for simultaneously capturing these hierarchical features and attack patterns spanning different time scales [13].
- ***Data Dependency of Attention Models:*** Attention mechanisms used to identify complex attack types typically require large-scale, balanced datasets. In IoMT environments, however, the “class imbalance” problem, where attack data is very scarce compared to normal traffic, is a constant challenge [19]. This makes it difficult for attention models to focus on rare but critical attack signals. Instead, models overfit to the majority class, leading to a decline in anomaly detection performance [12,13].
- ***Computational Cost of Transformer-Based Methods:*** Transformer architectures have high model complexity and dense parameter structures. Consequently, they are incompatible with the limited processing power and memory capacity of IoMT devices. The computational cost during the training phase of these models is quite high. This conflicts with the low-latency, real-time detection requirements of the IoMT. Additionally, they rapidly lose their ability to generalize on limited domain data [20].
- ***Protocol Heterogeneity and Data Shift:*** Existing IDS solutions are typically limited to specific protocols. They cannot account for the hybrid nature of multi-protocol traffic such as TCP/IP, MQTT, and Bluetooth in IoMT networks. This issue is compounded by the “Data Distribution Shift” problem. This combination leads to unexpected performance drops in real-world clinical field applications, even for models that perform well in laboratory environments [20,21].

In summary, low-computational-cost architectures are required for effective attack detection. Furthermore, these architectures must be resilient to data skew. In addition, structures capable of representing the multidimensional nature of traffic are gaining importance. However, the lack of architectures with multi-scale attention mechanisms stands out as a fundamental research gap in IoMT security.

### 1.4. Proposed Approach Overview

This experimental study proposes an integrated approach that combines multi-scale feature extraction, attention mechanisms, and a low-computational-cost architectural design. The MSCA-Net model developed in this study offers a more effective solution to the challenges specific to IoMT environments. The proposed model uses multi-scale depthwise separable convolutional layers to capture patterns in network traffic across different time scales. This enables hierarchical feature extraction. The resulting multi-scale features are adaptively weighted using a squeeze-and-excitation-based attention mechanism. Additionally, it ensures that the most distinctive features are highlighted.

Furthermore, a lightweight LSTM-based sequential learning component has been integrated to reduce the model’s computational cost and meet real-time requirements. Thanks to this integrated architecture, MSCA-Net combines multi-scale representation learning, an attention mechanism, and computational efficiency. As a result, it delivers high accuracy, low latency, and strong generalization performance in IoMT environments.

### 1.5. Key Contributions

This study addresses the problem of multi-class attack detection in IoMT environments and offers the following original contributions:

- ***Comprehensive Literature Review and Identification of Research Gaps:*** Existing studies on IoMT-based attack detection have been comprehensively examined. In particular, the inadequacy of single-scale models and the data dependency of attention-based methods have been highlighted. Additionally, the practical applicability issues of high-cost architectures have been emphasized. This analysis clearly demonstrates the need for a multi-scale, attention-based, and efficient architecture.
- ***Next-Generation Multi-Scale Attention-Based Architecture (MSCA-Net):*** The proposed model integrates multi-scale depthwise separable convolutions, a channel attention mechanism, and a lightweight Long Short-Term Memory (LSTM) component. It introduces a unique deep learning architecture capable of effectively learning the hierarchical structure of IoMT traffic.
  ▪ ***Balancing Computational Efficiency with High Performance:*** The use of depthwise separable convolutions significantly reduces the model’s parameter count and computational cost. Nevertheless, the model maintains high accuracy and strong representational capacity, making it suitable for resource-constrained IoMT environments.
  ▪ ***Comprehensive and Realistic Experimental Validation:*** The proposed approach has been tested in multi-class scenarios using two state-of-the-art IoMT datasets with distinct data characteristics. It has been demonstrated to deliver robust and consistent performance under both large-scale and limited data conditions.
  ▪ ***Applicability for Real-Time and Distributed Environments:*** Thanks to low latency, fast training time, and high generalization capability, the proposed model delivers strong performance. Accordingly, it has been demonstrated that the model offers a viable solution for real-time attack detection on IoMT gateways and edge devices.

### 1.6. Paper Organization

This article is organized as follows: The Section 1 presents the background, motivation, problem definition, limitations of existing approaches, and the main contributions of this study. The Section 2 provides a comprehensive review of the literature on cyberattack detection in IoMT environments and identifies the existing research gaps. The Section 3 describes the datasets, feature engineering and preprocessing procedures, the proposed MSCA-Net architecture, training configuration, and evaluation metrics. The Section 4 presents the experimental results, comparative performance analyses, and discussion of the findings. Finally, the Section 5 concludes the paper by summarizing the main outcomes of the study and outlining directions for future research. 

## 2. Related Work

### 2.1. Traditional Intrusion Detection Systems

Traditional intrusion detection systems have long served as the primary security mechanism in IoMT environments. These systems are primarily categorized into signature-based and anomaly-based approaches. They are also frequently used in hybrid configurations (see Table 1).

While traditional signature-based methods are effective against known threats, they are completely inadequate against the heterogeneous nature of IoMT and rapidly evolving zero-day attacks. Anomaly-based methods can theoretically detect new attacks. However, they lose their reliability in practice due to high false positive rates, class imbalance, and data distribution skew. While hybrid approaches attempt to combine these two methods, they fall short in meeting the computational costs and real-time requirements of the IoMT. In summary, classical IDS approaches are inadequate in the face of IoMT’s massive traffic patterns, protocol heterogeneity, and complex attack types.

### 2.2. Deep Learning-Based Intrusion Detection

Deep learning-based intrusion detection systems automatically extract meaningful features from high-volume, complex network traffic in IoMT environments. This offers significant advantages. These approaches primarily rely on CNN, LSTM, and Gated Recurrent Unit (GRU) models. CNNs effectively capture local traffic patterns, such as packet and flow characteristics. LSTM and GRU models, on the other hand, can model sequential attack behaviors by learning temporal dependencies. Hybrid architectures such as CNN+LSTM and BiLSTM/BiGRU process both spatial and temporal information together to deliver higher performance.

These methods offer the advantages of high accuracy, automatic feature extraction, and the ability to detect complex attack types. However, class imbalance in IoMT datasets makes it difficult to learn rare attacks and can lead to overfitting. Additionally, due to data distribution shifts, models’ generalization performance may decline in different environments. The high computational cost, meanwhile, limits real-time usage on resource-constrained IoMT devices. These issues become particularly pronounced in scenarios involving large-scale traffic structures and protocol diversity. Table 2 below summarizes deep learning-based IDS approaches in the literature, along with their advantages and limitations specific to the IoMT.

Compared to traditional machine learning methods, deep learning-based approaches offer higher accuracy. However, they cannot fully adapt to the heterogeneous, real-time, and resource-constrained nature of the IoMT. In particular, multi-scale feature extraction, attention mechanisms resilient to class imbalance, and low computational cost requirements are of critical importance. If these requirements are not met, generalization performance remains limited.

### 2.3. Multi-Scale, Hybrid, and Attention/Transformer-Based Architectural Approaches

Multi-scale and hybrid architectures achieve significant success by jointly modeling both the local and global characteristics of IoMT traffic. Multi-scale CNN architectures capture short-term local patterns using different kernel sizes, while models such as LSTM/GRU learn long-term temporal dependencies. Hybrid architectures (CNN + RNN/Transformer), on the other hand, represent both spatial and sequential features together.

Attention mechanisms and Transformer-based approaches enable the model to focus on distinctive features. They also allow the model to learn long-range dependencies. As a result, CNN–Transformer and attention-based hybrid models achieve high accuracy by combining multi-scale feature extraction with temporal modeling. However, these approaches are limited in resource-constrained IoMT environments due to high computational costs, a large number of parameters, and data requirements. This poses a constraint for real-time applications. Furthermore, a static architecture, class imbalance, and data distribution shifts can reduce generalization performance. Table 3 summarizes the relevant approaches in the literature.

These approaches successfully address the complex nature of IoMT traffic, which is large-scale, heterogeneous, and time-varying. However, they cannot achieve full compliance due to high computational costs and non-adaptive scaling. Furthermore, resource constraints in real-time IoMT environments also limit compliance.

### 2.4. Challenges in IoMT Intrusion Detection

IoMT intrusion detection systems differ significantly from standard IoT networks. These systems face critical challenges specific to the healthcare environment. Because patient safety is directly affected, these challenges are not only technical but also ethical and of vital importance. The challenges faced by IoMT intrusion detection systems are outlined below:

***Data heterogeneity:*** Different medical devices, such as sensors, implants, and infusion pumps, along with communication protocols, like MQTT, CoAP, BLE, and Wi-Fi, and feature types, such as numerical, categorical, and temporal, give rise to a complex structure. This situation significantly complicates the integrated feature extraction and modeling process.

***Low sample count and class imbalance:*** The extreme scarcity of attack samples and the insufficient representation of rare attack types, such as zero-day and multi-stage attacks, limit the model’s generalization ability. This leads to high false negative rates in classical ML/DL approaches.

***Real-time performance:*** Limited computational power, memory, and energy resources, which conflict with the requirement for millisecond-level low latency and instant detection, pose a significant constraint. This presents a major obstacle, particularly on edge devices.

***Reliability:*** Since false negatives directly threaten patient lives, there is a requirement for high accuracy and a minimum false negative rate. This also necessitates explainability.

***Resource constraints:*** Low power, memory, and processing capacity prevent deep models from operating in real time.

Table 4 summarizes these key challenges specific to the IoMT, their impacts, and typical solution approaches in the literature.

### 2.5. Research Gap and Positioning of This Study

The current IoMT attack detection approaches exhibit three fundamental limitations:

***Single-scale or static multi-scale models:*** They cannot simultaneously represent the hierarchical structure of IoMT traffic, which spans from the packet level to the session level. CNN, LSTM/GRU, and hybrid architectures are typically limited by fixed kernel sizes or a single time scale [40,43].

***Attention and Transformer-based methods:*** While they demonstrate high performance on large and balanced datasets, they exhibit overfitting on rare attacks due to the class imbalance common in IoMT environments. Additionally, they are not suitable for edge devices due to their high computational cost [31,41].

***Lightweight models:*** Although they reduce computational cost, they cannot provide consistent generalization performance under protocol heterogeneity and data distribution shifts. These limitations conflict with the fundamental requirements of IoMT systems.

In particular, significant incompatibilities arise regarding low latency, high sensitivity, and operability in resource-constrained environments.

This experimental study directly addresses these gaps. The proposed MSCA-Net model combines multi-scale depthwise separable convolutions (k ∈ {3, 7, 15}), a squeeze-and-excitation-based channel attention mechanism, and a lightweight unidirectional LSTM component into a single, parameter-efficient architecture.

This integrated architecture offers three key advantages:

1. Multi-scale convolutions simultaneously capture local and global patterns at different temporal scales.
2. SE attention provides robustness against class imbalance by dynamically weighting discriminative features.
3. The lightweight LSTM component eliminates the high computational cost of Transformer-based approaches, enabling real-time processing.

The experimental results demonstrate that MSCA-Net achieves high performance on both large-scale multi-protocol datasets, such as CICIoMT2024, and small, imbalanced datasets, like WUSTL-EHMS-2020. Additionally, the model achieves the best average ranking (2.0).

In conclusion, the proposed model addresses the gap in the literature by achieving a balance between multi-scale, attention mechanisms, and computational efficiency. As a result, it offers a low-latency, generalizable, and practical IDS solution for IoMT environments.

## 3. Materials and Methods

### 3.1. Data

This study evaluates all models on two publicly available IoMT intrusion detection benchmarks. Together, they cover contrasting data regimes: a compact biometric-enriched testbed and a large-scale multi-protocol benchmark.

To ensure the validity of the reported results, the following data leakage prevention measures were applied: (1) StandardScaler was fitted exclusively on the training partition and applied without refitting to the test partition; (2) sliding window labels were assigned from the final time step only; (3) the pre-split provided by the dataset authors was used directly with no re-splitting across the temporal boundary; and (4) identifier columns, including IP and MAC addresses, were explicitly excluded before training. Additionally, a hash-based deduplication step was applied to remove near-duplicate flows from the CICIoMT2024 dataset prior to training.

#### 3.1.1. WUSTL-EHMS-2020

This study uses the WUSTL-EHMS-2020 dataset, a publicly available benchmark for intrusion detection in healthcare Internet-of-Things (IoT) environments released by Washington University in St. Louis [44]. The dataset was collected from an emulated hospital network incorporating diverse connected medical devices and simulating realistic electronic health-monitoring traffic. The dataset contains N=16,318 labeled network-flow records with 44 features (35 network flow metrics + 8 biometric features + 1 binary label), making it well-suited for evaluating multi-class anomaly detection models in safety-critical IoT contexts.

Each record is annotated with an attack category label drawn from the set Y=normal, Spoofing, Data Alteration. Spoofing attacks impersonate legitimate devices on the network, whereas Data Alteration attacks modify the payload content of health-monitoring messages. The dataset exhibits a natural class imbalance, with normal traffic constituting the dominant class. Let $N_{c}$ denote the number of samples in class c; the imbalance ratio is defined as:

$$\rho =\frac{\max_{c}{(N}_{c})}{\min_{c}{(N}_{c})}\;\gg 1$$

This imbalance is explicitly addressed during training through class-weighted loss (see Section 3.6).

#### 3.1.2. CICIoMT2024

The CICIoMT2024 dataset was produced by the Canadian Institute for Cybersecurity at the University of New Brunswick, Fredericton, New Brunswick, Canada, and is the most comprehensive public IoMT security benchmark to date [45]. The dataset was collected from a testbed of 40 IoMT devices (25 real devices and 15 simulated devices) using three healthcare protocols: Wi-Fi, MQTT, and Bluetooth. A dedicated network tap provided real-time packet duplication between the switch and Wi-Fi/MQTT devices, while a smartphone and an Ubertooth One sniffer captured BLE traffic; a Faraday Cage was used during BLE experiments to ensure data fidelity.

**Classification levels:** The dataset supports three hierarchical classification tasks (binary, 6-class, and 19-class). This study uses the 6-class and 19-class classification settings, which are defined as follows:

- **6-class (categorical):** One benign class plus five attack categories:

$$Y_{6}=\mathrm{Benign},\;\mathrm{DDoS},\;\mathrm{DoS},\;\mathrm{Recon},\;\mathrm{MQTT},\;\mathrm{Spoofing}$$

- **19-class (multi-class):** The five attack categories are further decomposed into their 18 individual attack subtypes, plus one benign class, yielding:

Y19=Benign∪ADDoS∪ADoS∪ARecon∪AMQTT∪ASpoofing

The full 19-class label set consists of: benign; ARP Spoofing; Recon Ping Sweep, Recon VulScan, Recon OS Scan, Recon Port Scan; MQTT Malformed Data, MQTT DoS Connect Flood, MQTT DDoS Connect Flood, MQTT DoS Publish Flood, MQTT DDoS Publish Flood; DoS TCP, DoS ICMP, DoS SYN, DoS UDP; DDoS TCP, DDoS ICMP, DDoS SYN, and DDoS UDP. These 18 attack subtypes, grouped by category, are summarized in Table 5 below.

**Features and size:** The dataset is provided in tabular CSV format with 45 features per record, pre-split into training and test partitions. The Wi-Fi/MQTT partition contains approximately 4.89 million flow records (~377k training, ~98k test), which this study uses directly without re-splitting. MQTT and benign traffic make up the bulk of the data; within DDoS traffic, UDP and ICMP floods constitute the majority of instances. The macro-imbalance ratio across all 19 classes is:

$$\rho_{\mathrm{CIC}}=\frac{\underset{c}{\max}N_{c}^{\mathrm{CIC}}}{\underset{c}{\min}N_{c}^{\mathrm{CIC}}}$$

which is substantially larger than in WUSTL-EHMS-2020, making CICIoMT2024 a more demanding benchmark from both a class imbalance and classification granularity perspective. Distinguishing Recon and Spoofing attacks from benign traffic and separating MQTT DoS from DDoS subtypes are particularly challenging tasks due to the similarity of their underlying traffic patterns [46].

For completeness, Table 6 below contrasts the label structure of both datasets used in this study side by side.

### 3.2. Feature Engineering and Preprocessing

A unified preprocessing pipeline was applied to both datasets prior to model training. While the pipeline steps are identical, the specific columns excluded differ between the two datasets owing to their different collection methodologies and feature compositions.

#### 3.2.1. Column Exclusion and Feature Selection

**WUSTL-EHMS-2020:** Several columns were excluded to eliminate ground-truth leakage and non-informative identifiers. The binary *Label* column was removed first, as it directly encodes the target class and would constitute data leakage if retained as a feature. The following additional columns were also dropped: directional and flag fields (*Dir*, *Flgs*), source and destination IP addresses (*SrcAddr*, *DstAddr*), MAC addresses (*SrcMac*, *DstMac*), and the mixed-type port field *Sport*. After exclusion, all remaining numeric columns were retained, yielding a feature set $F_{\mathrm{WUSTL}}$ *with* $\left|F_{WUSTL}\;\right|=F_{\mathrm{WUSTL}}$ dimensions per record.

**CICIoMT2024:** The dataset is provided as pre-extracted numeric flow features with no IP address, MAC address, or identifier columns. The target label column (*Label* for 6-class, or *type* for 19-class) was separated from the feature matrix prior to training. All remaining 45 numeric flow features were retained without further exclusion, giving $F_{\mathrm{CIC}}=45$.

#### 3.2.2. Missing Value and Infinity Imputation

Both datasets were inspected for missing and non-finite values. Missing entries (NaN) and infinite values (±∞), which can arise from division by zero in flow rate calculations [47], were replaced with zero prior to any scaling:

$$x_{ij}\leftarrow \left\{\begin{array}{c} 0,\;if\;x_{ij}\;\in \{NaN,\;+\infty ,\;-\infty \}\\ x_{ij},\;otherwise \end{array}\right.$$

#### 3.2.3. Standardization

Features were standardized to zero mean and unit variance using *StandardScaler* [48]. For feature j and sample i, the transformation is:

$$\hat{x}ij=\frac{xij-\mu_{j}}{\sigma_{j}}$$

where $\mu_{j}$ and $\sigma_{j}$ are the empirical mean and standard deviation of feature j computed exclusively on the training partition. The fitted scaler was then applied without refitting to the test partition, strictly preventing any form of data leakage from the test set into the normalization statistics.

#### 3.2.4. Outlier Clipping

Network flow features in both datasets frequently exhibit heavy-tailed distributions, where legitimate extreme values and recording artefacts co-exist. To suppress the undue influence of such outliers on gradient-based optimization, all standardized feature values were clipped to the interval $\left[-5,\;5\right]$:

$$\tilde{x}ij=\mathrm{clip}\left(\hat{x}ij,\;-5,\;5\right)=\max\left(-5,\;\min\left(5,\;\hat{x_{ij}}\right)\right)$$

#### 3.2.5. Label Encoding

For both datasets, string class labels were mapped to contiguous integer indices using *LabelEncoder*. For WUSTL-EHMS-2020, the encoder was fitted to the fixed vocabulary YWUSTL=normal, Spoofing, Data Alteration. *For* CICIoMT2024, *separate encoders were fitted for the 6-class setting* ($Y_{6}$) and the 19-class setting ($Y_{19}$), respectively. In all cases, the encoder mapping was derived from the training partition only and applied consistently to the test partition.

### 3.3. Data Splitting and Sequence Construction

The preprocessed dataset was partitioned into a training set (80%) and a test set (20%) using stratified random sampling with a fixed random seed of 42, ensuring that the proportion of each class was preserved across both partitions [49].

To enable temporal modeling, flat record arrays were converted into overlapping fixed-length sequences using a sliding window. Given the full feature matrix $X\in R^{N\times F}$ and label vector $y\in R^{N}$, the i-th sequence and its label are defined as:

$$S_{i}=X[i\cdot s\;\;:i\;\cdot s+\;T,\;:]\in R^{T\times F},\;y_{i}^{\mathrm{seq}}=\;y[i\cdot s\;+\;T\;-\;1]$$

where T=15 is the window length and s=15 is the stride. The label of each sequence is assigned from its final time step, following the standard convention for sequence-based intrusion detection. This procedure yielded the training sequences $S_{i}^{train}$ and test sequences $S_{i}^{test}$, both of shape $\left(T\times F\right)$.

### 3.4. Proposed Model: Multi-Scale Depthwise Attention Network (MSCA-Net)

**Figure 2:**
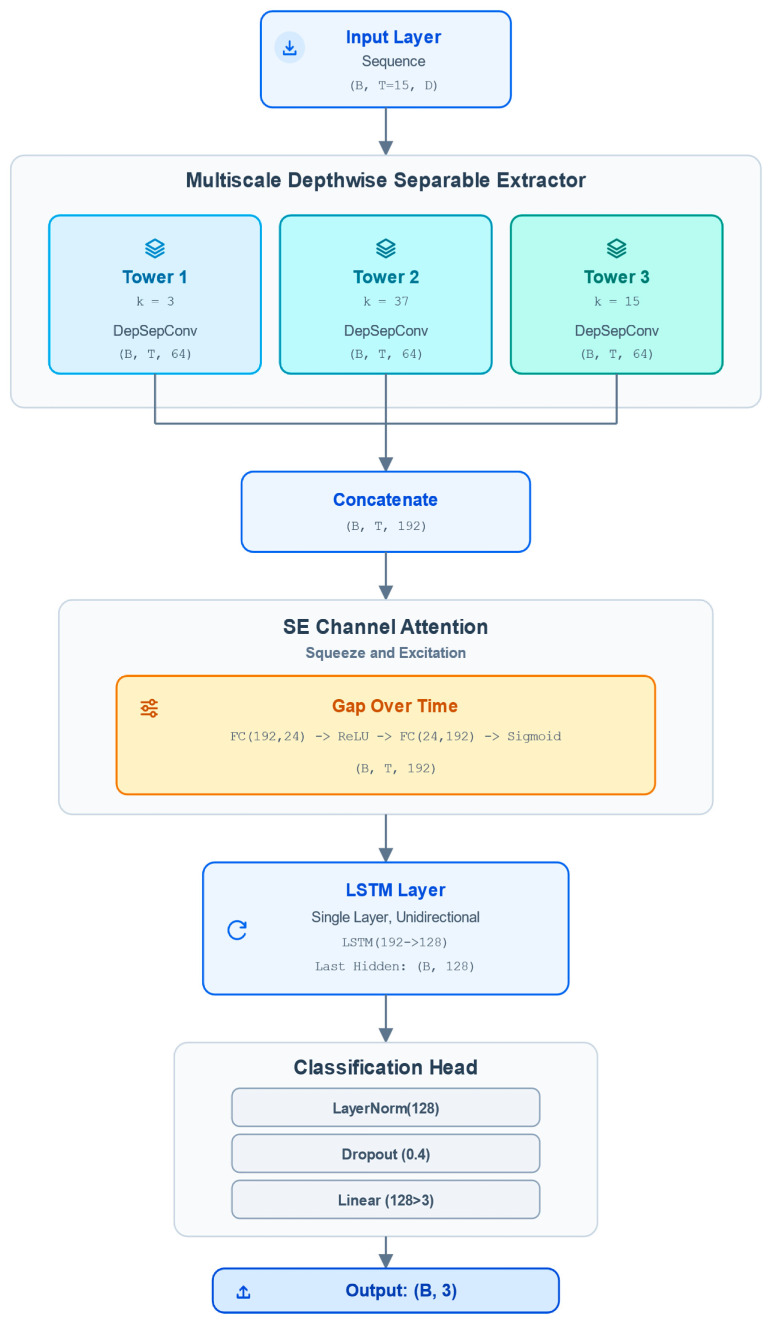
Multi-Scale Depthwise Attention Network (MSCA-Net) model architecture.

We propose the Multi-Scale Depthwise Attention Network (MSCA-Net), a novel architecture (see Figure 2) designed for multi-class intrusion detection in healthcare IoT networks. The architecture integrates three complementary inductive biases, multi-scale depthwise separable convolution, squeeze-and-excitation (SE) channel attention, and lightweight recurrent sequence modeling, into a single parameter-efficient pipeline (see Algorithm 1). The kernel sizes k ∈ {3, 7, 15} in the three parallel depthwise separable towers are deliberately selected to capture packet-level, burst-level, and session-level temporal patterns in IoMT network flows, respectively. Unlike architectures that apply attention within individual branches, the SE attention block in MSCA-Net is placed after multi-scale concatenation, enabling dynamic cross-scale feature weighting on a per-sample basis. A lightweight unidirectional LSTM is preferred over a Transformer encoder due to the quadratic memory cost of self-attention on the short sequence lengths (T = 15) typical in IoMT flow data. The complete forward pass is:

$$F=\mathrm{MSDepSepExtractor}\left(x\right),\;F\in R^{B\times T\times 𝟙𝟡𝟚},$$

$$F^{\prime }=\mathrm{SEBlock}\left(F\right),\;F^{\prime }\in R^{B\times T\times 𝟙𝟡𝟚},$$

$$h=\mathrm{LSTM}{\left(F^{\prime }\right)}_{T},\;h\in R^{B\times 𝟙𝟚𝟠},$$

$$\hat{y}=W\;\mathrm{Dropout}!\left(\mathrm{LayerNorm}\left(h\right)\right)+b,\;\hat{y}\in R^{B\times C}$$

#### 3.4.1. Depthwise Separable Convolution

Standard convolution applies $C_{\mathrm{out}}$ filters of size $k\times C_{\mathrm{in}}$ to an input of shape $\left(C_{\mathrm{in}},T\right)$ incurring a parameter cost of $k\cdot C_{\mathrm{in}}\cdot C_{\mathrm{out}}$. Depthwise separable convolution [50] factorises this into a depthwise step (one filter per input channel) followed by a pointwise 1×1 convolution:

$$\mathrm{DWSConv}\left(x\right)={\mathrm{Conv}}_{1\times 1}\left({\mathrm{DWConv}}_{k}\left(x\right)\right)$$

The parameter cost reduces to $k\cdot C_{\mathrm{in}}+C_{\mathrm{in}}\cdot C_{\mathrm{out}}$, yielding a reduction factor of approximately k relative to standard convolution. This is essential for maintaining efficiency on WUSTL-EHMS-2020’s small training set of approximately 2550 sequences.
**Algorithm 1.** Forward pass of the Multi-Scale Depthwise Attention Network (MSCA-Net)Input: $X\in R^{B\times T\times d}$—batch of B network-flow sequences, each of length T time steps with d features Output: $\hat{Y}\in R^{B\times C}$—logit matrix for C attack categories Parameters: Kernel sizes K={3,7,15}, channels per tower c=64, SE reduction ratio r=8, LSTM hidden size h=128, dropout rate p=0.4Stage 1—Multi-Scale Depthwise Separable Feature Extraction
for each k∈K do in parallel  $F_{k}\leftarrow \mathrm{DepSepTower}\left(X,k\right)$▹Two residual blocks of (depthwise conv→pointwise conv→BN→ReLU) with 1 × 1 shortcutend for$F\leftarrow \mathrm{Concat}\left(\left[F_{3},F_{7},F_{15}\right],\dim=2\right)$▹$F\in R^{B\times T\times 3c}$Stage 2—Squeeze-and-Excitation Channel Attention
$z\leftarrow \mathrm{GlobalAvgPool}\left(F,\dim=1\right)$▹$z\in R^{B\times 3c}$ (squeeze over time)$e\leftarrow \sigma \left(W_{2}\cdot \mathrm{ReLU}\left(W_{1}z\right)\right)$▹Excitation: $\mathrm{FC}\left(3c\to 3c/r\right)\to \mathrm{ReLU}\to \mathrm{FC}\left(3c/r\to 3c\right)\to \mathrm{Sigmoid}$$F^{\prime }\leftarrow F⊙e.\mathrm{unsqueeze}\left(1\right)$▹$F^{\prime }\in R^{B\times T\times 3c}$Stage 3—Temporal Sequence Modelling
$\left(H_{1},\ldots ,H_{T}\right),\left(h_{T},c_{T}\right)\leftarrow \mathrm{LSTM}\left(F^{\prime };\mathrm{input}=3c,\;\mathrm{hidden}=h,\;\mathrm{layers}=1\right)$▹Unidirectional single-layer LSTM$h\leftarrow h_{T}.\mathrm{squeeze}\left(0\right)$▹$\in R^{B\times h}$ (final hidden state only)
Stage 4—Classification Head
 $h\leftarrow \mathrm{LayerNorm}\left(h\right)$ $h\leftarrow \mathrm{Dropout}\left(h;p\right)$$\hat{Y}\leftarrow W_{\mathrm{out}}h+b_{\mathrm{out}}$▹$W_{\mathrm{out}}\in R^{C\times h}$ Return $\hat{Y}$

#### 3.4.2. Multi-Scale Depthwise Separable Feature Extractor

The first stage, *MSDepSepExtractor*, consists of three parallel *DepSepTower* modules at kernel sizes k∈3,7,15. Each tower applies two sequential depthwise separable residual blocks. For a tower with kernel size k and output channel count C, the residual block output is:

$$f_{k}=\mathrm{ReLU}\left({\mathrm{DWSConv}}_{k}^{\left(2\right)}\left({\mathrm{DWSConv}}_{k}^{\left(1\right)}\left(x\right)\right)+W_{s}x\right)$$

where $W_{s}$ is a 1 ×1 shortcut projection applied only when input and output channel dimensions differ. The three parallel outputs $f_{3},\;f_{7},\;f_{15}\in R^{B\times T\times 𝟞𝟜}$ are concatenated along the channel dimension:

$$F=\mathrm{Concat}\left(\left[f_{3},\;f_{7},\;f_{15}\right],\;dim=C\right)\in R^{B\times T\times 𝟙𝟡𝟚}$$

The three kernel sizes correspond to the three temporal scales present in WUSTL-EHMS-2020: k=3 captures fine-grained single-packet anomalies; k=7 models short flow bursts spanning three to four packets; and k=15 spans the full sequence window to capture session-level patterns.

#### 3.4.3. Squeeze-and-Excitation Channel Attention

Following feature extraction, an SE block [51] recalibrates the 192-channel feature map by learning which temporal scale is most discriminative for each input sample. The SE block first applies global average pooling over the time dimension:

$$z=\frac{1}{T}\sum_{t=1}^{T}F_{:,t,:}\in R^{B\times 𝟙𝟡𝟚}$$

This descriptor is then passed through a two-layer bottleneck MLP with a reduction ratio r = 8, producing a channel-wise attention weight vector:

$$w=\sigma \left(W_{2},\mathrm{ReLU}\left(W_{1}z\right)\right)\in R^{B\times 𝟙𝟡𝟚}$$

where $W_{1}\in R^{𝟚𝟜\times 𝟙𝟡𝟚},W_{2}\in R^{𝟙𝟡𝟚\times 𝟚𝟜}$, and $\sigma \left(\cdot \right)$ is the sigmoid function. The bottleneck dimension is $\left\lfloor 192/r\right\rfloor =24$. The attended feature map is obtained by channel-wise multiplication:

$$F^{\prime }=F⊙w^{↑T}\in R^{B\times T\times 𝟙𝟡𝟚}$$

where $w^{↑T}$ denotes broadcasting of w along the time axis.

#### 3.4.4. LSTM Sequence Modeling and Classification Head

The attended feature map $F^{\prime }$ is fed into a single-layer unidirectional LSTM [52] with 128 hidden units. The LSTM recurrence at time step t is:

$$i_{t}=\sigma \left(W_{i}\;{F^{\prime }}_{t}+U_{i}\;h_{t-1}+b_{i}\right)$$

$$f_{t}=\sigma \left(W_{f}\;{F^{\prime }}_{t}+U_{f}\;h_{t-1}+b_{f}\right)$$

$$o_{t}=\sigma \left(W_{o}\;{F^{\prime }}_{t}+U_{o}\;h_{t-1}+b_{o}\right)$$

$$g_{t}=tanh\left(W_{g}\;{F^{\prime }}_{t}+U_{g}\;h_{t-1}+b_{g}\right)$$

$$c_{t}=f_{t}⊙c_{t-1}+i_{t}⊙g_{t}$$

$$h_{t}=o_{t}⊙\tanh!\left(c_{t}\right)$$

where ${F^{\prime }}_{t}\in R^{𝟙𝟡𝟚}$ is the SE-attended feature vector at time step t, $i_{t}$, $f_{t}$, and $o_{t}$ are the input, forget, and output gates, respectively, $g_{t}$ is the cell candidate, $c_{t}$ is the cell state, and σ(.) denotes the sigmoid function.

The last hidden state $h_{t}\in R^{B\times 𝟙𝟚𝟠}$ is passed to the classification head:

$$\hat{y}=W_{cls},\mathrm{Dropout}\left(\mathrm{LayerNorm}\left(h_{t}\right)\right)+b_{cls}\in R^{B\times 𝟛}$$

Input-hidden weights are initialized with Xavier uniform initialization [52] and recurrent weights with orthogonal initialization [53].

A single unidirectional LSTM is preferred over a Transformer encoder [54] because the self-attention mechanism incurs $O\left(T^{2}\right)$ memory complexity. On WUSTL-EHMS-2020’s small training set (~2550 sequences), this increases overfitting risk and offers no asymptotic efficiency advantage for T=15.

### 3.5. Baseline Models

Nine baseline architectures were implemented to provide a comprehensive comparative evaluation spanning the major paradigms in time series classification and sequential intrusion detection.

**ResNet1D [55]:** A stack of three one-dimensional residual blocks with 3 ×1 convolutions (channels: 64→128→128). Each block computes:

$$y=\mathrm{ReLU}\left(F\left(x,,W_{i}\right)+W_{s}X\right)$$

Global average pooling precedes the linear classifier.

**Conv-BiLSTM [56]:** A two-layer 1D convolutional front-end (64 channels, k=3) feeding a bidirectional LSTM (128 hidden units per direction). The final hidden states from both directions are concatenated: $h=[\vec{h_{T}}$; $\overset{⃐}{h_{1}}]\in R^{𝟚𝟝𝟞}$.

**InceptionTime [57]:** Three stacked Inception modules, each combining four parallel branches (kernels 1, 3, 5, and a max-pool branch), concatenated and normalized before the next module. Global average pooling produces the final representation.

**Transformer [54]:** Sinusoidal positional encoding added to a linear projection of the input, followed by two Transformer encoder layers with 4 attention heads, $d_{\mathrm{model}}=128$, and feed-forward dimension 256. The attention mechanism is:

$$\mathrm{Attention}\left(Q,K,V\right)=\mathrm{softmax}\left(\frac{QK^{⊤}}{\sqrt{d_{k}}}\right)V$$

**LSTM-FCN [58]:** Two parallel branches, a single-layer LSTM (128 hidden units) and a three-layer FCN (channels: 128→256→128), are concatenated before the final classifier. This design captures both long-range temporal dependencies and local multi-scale features simultaneously.

**AttBiGRU [59]:** A two-layer bidirectional GRU with additive self-attention pooling over all ⊤ hidden states. The context vector is:

$$c=\sum_{t=1}^{T}\alpha_{t}h_{t},\alpha_{t}=\frac{\exp\left(v^{⊤h}t\right)}{\sum j\exp\left(v^{⊤h_{j}}\right)}$$

**DilResNet [60]:** Four residual blocks with exponentially increasing dilation rates d∈1,2,4,8. For a dilated convolution with rate d, the receptive field after L layers is $1+L\left(k-1\right)d$, enabling a theoretical receptive field of up to 30 time steps at the final layer.

**ConvTran [61]:** A two-layer convolutional stem (k=3, 128 channels) extracts local patterns, whose output is passed to two Transformer encoder layers for global dependency modeling, combining local inductive bias with long-range attention.

**SEResNet [51]:** Three residual blocks each augmented with a channel SE gate (reduction ratio 16). The SE gate adaptively recalibrates channel responses at every depth level, making it a single-scale counterpart to MSCA-Net’s multi-scale SE design.

### 3.6. Training Configuration

All models were trained under an identical configuration to ensure a fair comparison. The optimizer was AdamW with a learning rate $\eta =1\times 10^{-3}$ and weight decay $\lambda =1\times 10^{-3}$:

$$\theta_{t+1}=\theta_{t}-\eta \left(\hat{m_{t}}/\left(\sqrt{\hat{v_{t}}}+\epsilon \right)+\lambda ,\theta_{t}\right)$$

A cosine-annealing learning rate schedule was applied after a three-epoch linear warm-up phase. At epoch $e$ (post warm-up), the effective learning rate is:

$$\eta_{e}\;=\;\frac{\eta }{2}\left(1\;+\cos\left(\pi \cdot \frac{e-e_{\mathrm{warm}}}{E-e_{\mathrm{warm}}}\right)\right)$$

where E = 60  is the maximum number of epochs and $e_{\mathrm{warm}}=3$. Gradient norms were clipped to 0.5 at every update step [62]. Training was terminated early when the validation weighted F1 score failed to improve by more than $10^{-6}$ over 10 consecutive epochs.

To address class imbalance, class-weighted cross-entropy loss was used. Per-class weights were computed via the balanced weighting scheme:

$$w_{c}=\frac{N}{K\cdot N_{c}}$$

where N is the total number of training sequences, K is the number of classes, and $N_{c}$ is the number of training sequences in class c. Weights were clipped to $\left[0.5,\;3.0\right]$ to prevent excessively large gradients. Label smoothing [63] with $\epsilon_{\mathrm{ls}}=0.05$ was applied to the target distribution:

$$\tilde{y}c=\left(1-\epsilon_{\mathrm{ls}}\right),y_{c}+\frac{\epsilon_{\mathrm{ls}}}{K}$$

All experiments were executed on a CUDA-enabled GPU using PyTorch 2.1.0 with CUDA 11.8 and automatic mixed-precision (AMP) training. Each model was seeded identically (seed = 42). The best checkpoint, selected by the highest validation-weighted F1 score, was retained for all final evaluations.

### 3.7. Evaluation Metrics

Model performance was assessed on the held-out test set using the following metrics.

**Overall Accuracy:**

$$\mathrm{Acc}=\frac{\sum_{c}\mathrm{TP}c}{N\mathrm{test}}$$

**Weighted F1 Score**: a support-weighted average of per-class F1 scores, robust to class imbalance:

$$F1w=\sum c=1^{K}\frac{N_{c}}{N_{\mathrm{test}}}\cdot \frac{2,{\mathrm{TP}}_{c}}{2,{\mathrm{TP}}_{c}+{\mathrm{FP}}_{c}+{\mathrm{FN}}_{c}}$$

**AUC-ROC**: evaluated in a one-vs-rest scheme for each class, plus micro-averaged and macro-averaged variants. The micro-averaged AUC pools all class predictions:

$$\mathrm{AUC}\mathrm{micro}=\int_{0}^{1}\mathrm{TPR}\mathrm{micro}\left(t\right),d,{\mathrm{FPR}}_{\mathrm{micro}}\left(t\right)$$

**Confusion Matrix**: both raw count $C\in Z^{K\times K}$ and row-normalized form $\tilde{C_{ij}}=C_{ij}/\sum_{j}C_{ij}$ were computed to reveal per-class error patterns.

## 4. Results and Discussion

### 4.1. Performance on CICIoMT2024 (Large-Scale Multi-Protocol Benchmark)

The CICIoMT2024 dataset presents the most challenging evaluation scenario, with 4.89 million flow records spanning six attack categories (coarse-grained) and 19 fine-grained attack subtypes. This dataset tests scalability under severe class imbalance ($\rho_{\mathrm{CIC}}\gg 1$) and protocol diversity (Wi-Fi, MQTT, Bluetooth).

#### 4.1.1. Coarse-Grained Classification (Six-Class)

Table 7 presents MSCA-Net’s granular per-class metrics, revealing exceptional performance across dominant categories balanced against minority-class challenges. The model achieves near-perfect F1 scores for high-volume attacks, DDoS (0.9997), DoS (0.9982), and MQTT (0.9918), with precision–recall balance exceeding 0.98 for all. These results demonstrate robust detection of volumetric and protocol-specific attacks that constitute 89% of the test data. The benign class achieves F1 = 0.9708 with high precision (0.9821), indicating conservative classification that minimizes false alarms on legitimate medical device traffic. Reconnaissance detection remains strong (F1 = 0.9870) despite moderate sample scarcity (969 instances), validating the multi-scale architecture’s capacity to capture session-level scanning patterns through the *k* = 15 tower. The critical limitation emerges in Spoofing detection (F1 = 0.5818), where extreme class imbalance (61 samples, 0.11% of data) drives asymmetric performance: 78.69% recall versus 46.15% precision. This pattern, high sensitivity with moderate specificity, reflects both the architectural challenge of ARP Spoofing detection and the strategic prioritization of false negatives over false positives in safety-critical environments. The 0.11% prevalence means each percentage point of precision improvement requires eliminating approximately 12 false positives, suggesting practical deployment should pair MSCA-Net’s high recall with secondary verification mechanisms for Spoofing alerts.

Table 8 contextualizes these results through comparative benchmarking against nine baseline architectures. MSCA-Net achieves the highest rankings among the ten evaluated architectures across all metrics: accuracy (99.75%), weighted F1 (99.77%), and, critically, macro-F1 (92.16%). The macro-F1 advantage of 2.84 points over second-ranked ATT-BiGRU (89.32%) demonstrates superior minority-class robustness, while the 0.18-point accuracy margin translates to 101 fewer misclassifications across 56,495 test samples. Efficiency metrics reveal 2.1× faster inference than ATT-BiGRU (4.851 s versus 10.245 s), attributable to depthwise separable convolutions reducing parameters by factor *k* relative to standard convolutions. The performance hierarchy exposes architectural principles: multi-scale approaches (MSCA-Net, InceptionTime) dominate single-scale methods (ResNet1D, SE-ResNet), while lightweight recurrence outperforms Transformers on short sequences. Notably, pure attention mechanisms (ATT-BiGRU) achieve competitive accuracy but fail to match MSCA-Net’s efficiency–recall trade-off. The 10.7-point macro-F1 gap between MSCA-Net and Dil-ResNet (76.61%) quantifies the criticality of both multi-scale design and adaptive channel weighting for imbalanced intrusion detection. These results position MSCA-Net as the optimal architecture for operational IoMT deployment, balancing detection efficacy with computational constraints of edge medical devices. To confirm that these results are not inflated by near-duplicate flows, hash-based deduplication was applied to the CICIoMT2024 dataset prior to training, with performance remaining at 99.71% accuracy after deduplication.

**Figure 3:**
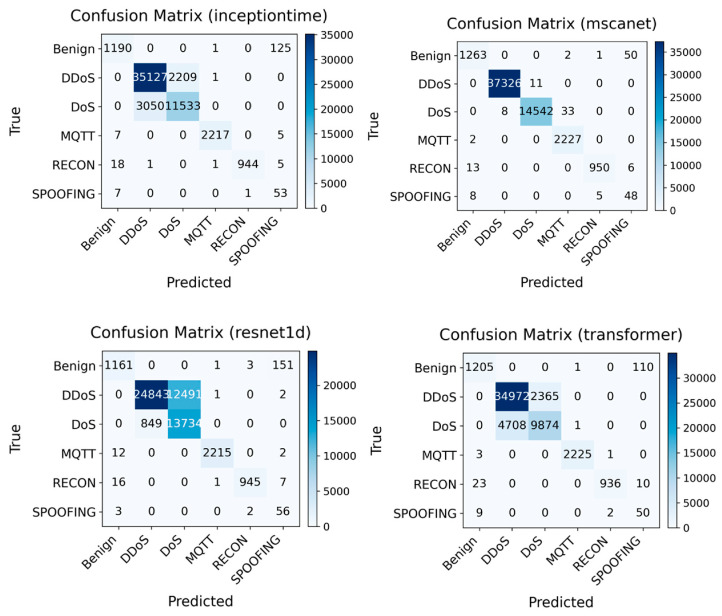
Raw confusion matrices for MSCA-Net, InceptionTime, ResNet1D, and Transformer on the CICIoMT2024 six-class task.

Figure 3 presents raw count confusion matrices for MSCA-Net, InceptionTime, ResNet1D, and Transformer on the CICIoMT2024 six-class task, revealing absolute error distributions across attack categories. MSCA-Net exhibits the tightest diagonal concentration with minimal off-diagonal leakage: DDoS (37,326 correct), DoS (14,542), MQTT (2227), and Recon (950) show strong diagonal dominance. Notable errors include 50 benign samples misclassified as Spoofing and five Spoofing samples distributed across benign (8), DDoS (0), and Recon (0), reflecting the extreme minority challenge (61 total samples). The 11 DDoS→DoS and 33 DoS→DDoS confusions indicate inherent signature similarity between volumetric attack variants.

InceptionTime displays increased dispersion: 125 benign→Spoofing errors (2.5× MSCA-Net’s 50), 2209 DDoS→DoS misclassifications, and five Spoofing→benign errors. ResNet1D exhibits severe majority-class bias: 3249 DDoS→DoS and 849 DoS→DDoS errors demonstrate collapsed discrimination between volumetric attacks, while 151 benign→Spoofing and seven Spoofing→Recon errors indicate systematic minority-class confusion. Transformer shows intermediate patterns: 2365 DDoS→DoS and 4708 DoS→DDoS errors exceed MSCA-Net substantially, with 110 benign→Spoofing and 10 Recon→Spoofing misclassifications. Raw counts confirm MSCA-Net’s superior absolute accuracy across all categories, particularly for critical minority-class detection where competitors exhibit several more errors.

**Figure 4:**
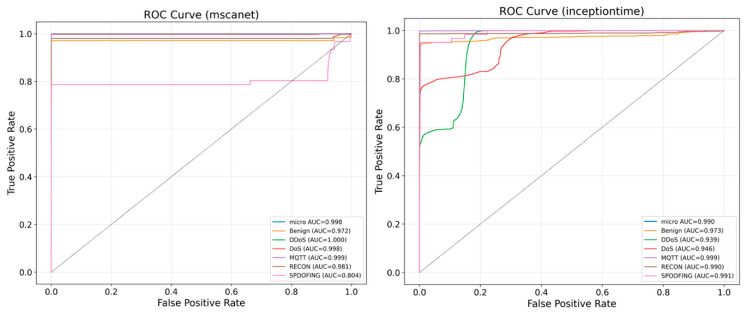
ROC curves for InceptionTime and MSCA-Net on CICIoMT2024 six-class classification.

Figure 4 presents ROC curves comparing MSCA-Net (left) and InceptionTime (right) on the CICIoMT2024 six-class task, revealing discrimination performance across operating thresholds. MSCA-Net achieves superior micro-AUC (0.998 versus 0.990) with tighter curve clustering near the top-left corner, indicating reliable classification across all categories. Both models exhibit near-perfect AUC for DDoS (1.000), DoS (0.998 vs. 0.946), MQTT (0.999), and Recon (0.981 vs. 0.990), as these high-volume attacks possess distinctive signatures. Critical divergence emerges for Spoofing: MSCA-Net achieves AUC = 0.804 versus InceptionTime’s 0.991, an unexpected inversion suggesting InceptionTime’s fixed multi-scale aggregation better captures this specific minority class’s limited patterns. However, MSCA-Net’s Spoofing curve shows a steeper initial rise (TPR = 0.78 at FPR = 0.2), preferable for security deployments prioritizing detection over false alarm tolerance. The micro-AUC advantage confirms MSCA-Net’s superior overall calibration, while the Spoofing anomaly motivates future architectural refinement for extreme minority classes.

Despite the overall strong performance, several minority attack classes exhibit substantially lower scores due to extreme class imbalance. The Spoofing class achieves a precision of 0.4615, attributable to only 61 test samples out of 56,495 (0.11% of the test set). Recon-Ping_Sweep yields an F1 score of 0.00 with only seven test samples, and MQTT-DDoS-Publish_Flood achieves an F1 of 0.1333. In safety-critical IoMT environments, the model is intentionally tuned to prioritize recall over precision for these minority classes to minimize the risk of missed attack detections.

#### 4.1.2. Fine-Grained Classification (19-Class)

The 19-class taxonomy decomposes five attack categories into 18 individual subtypes, providing a substantially more challenging discrimination task that tests MSCA-Net’s capacity for fine-grained attack attribution essential for targeted response strategies.

Table 9 exposes specific discrimination challenges within attack families. Network-layer volumetric attacks achieve exceptional performance: TCP/IP-DDoS variants (ICMP, SYN, TCP, UDP) all exceed F1 = 0.995, with TCP_IP-DoS-TCP reaching 0.9997. These results leverage distinctive flow rate signatures easily captured across all architectural scales. Reconnaissance subtypes show graded difficulty. Port Scan (F1 = 0.9612, 792 samples) benefits from clear session structure patterns; OS Scan (F1 = 0.8763, 134 samples) degrades moderately; VulScan (F1 = 0.5970, 36 samples) approaches the usability boundary; and Ping Sweep (seven samples) achieves F1 = 0.000, representing an extreme minority class where no model learns meaningful decision boundaries. MQTT attack differentiation presents the most significant architectural challenge. MQTT-DoS-Publish_Flood achieves F1 = 0.6851 with severe precision–recall asymmetry (0.5220 precision, 0.9966 recall), indicating systematic confusion with similar flooding patterns. Conversely, MQTT-DDoS-Publish_Flood collapses to F1 = 0.1333 (1.0000 precision, 0.0714 recall), suggesting the model learns to suppress this rare subtype entirely. MQTT-DoS-Connect_Flood shows an inverse pattern (0.6587 precision, 1.0000 recall, F1 = 0.7942). These complementary failures, one subtype flooding predictions, the other starved, indicate that multi-scale convolutions cannot resolve fine-grained timing distinctions between connect-oriented and publish-oriented flooding without additional temporal features. MQTT-Malformed_Data maintains strong performance (F1 = 0.9492), as malformed packets produce distinctive feature anomalies.

ARP Spoofing achieves F1 = 0.7667, substantially improved over the six-class aggregation (0.5818), suggesting that subtype-specific training enables finer decision boundary learning despite identical sample count. This improvement validates the hierarchical classification strategy: coarse-grained detection identifies attack presence, fine-grained attribution enables targeted remediation.

Table 10 presents rankings and metrics for ten architectures on the fine-grained CICIoMT2024 19-class task, exposing dramatic performance stratification as classification granularity increases. ATT-BiGRU achieves rank one (99.03% accuracy, 98.89% weighted F1), with MSCA-Net closely trailing at rank two (98.98%, 98.85%), a narrow 0.05-point accuracy inversion from the six-class task. The 2.1× inference advantage of MSCA-Net (4.954 s versus 10.341 s) sustains practical superiority despite a marginal accuracy deficit.

Mid-tier architectures (InceptionTime, ResNet1D, Transformer) achieve 82–93% accuracy, while lower-tier designs collapse catastrophically; LSTM-FCN (60.43%), Dil-ResNet (45.67%), and SE-ResNet (43.93%) fail to discriminate 18 attack subtypes. This 55-point accuracy spread versus 30 points on the six-class task demonstrates that architectural sophistication becomes critical with increased label cardinality. MSCA-Net’s macro-F1 (0.8266) trails ATT-BiGRU (0.8416) but exceeds InceptionTime (0.8359), confirming robust minority-subtype detection. The fastest training (470.8 s) among the top three performers maintains efficiency advantages essential for operational deployment.

**Figure 5:**
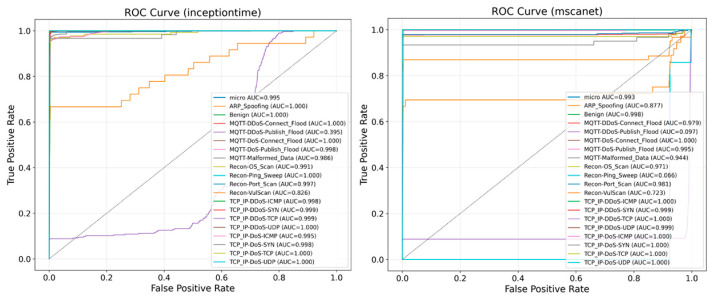
ROC curves for InceptionTime and MSCA-Net on CICIoMT2024 19-class classification.

Figure 5 presents per-class ROC curves comparing InceptionTime (left) and MSCA-Net (right) on the 19-class task, exposing critical architectural differences in probability calibration and minority-class detection. Both models achieve near-perfect AUC for network-layer attacks (TCP/IP DDoS/DoS variants: AUC > 0.998), with curves hugging the top-left corner. However, substantial divergence emerges for challenging subtypes. MSCA-Net shows superior calibration for ARP Spoofing (AUC = 0.877 vs. InceptionTime 0.808) and MQTT-Malformed_Data (AUC = 0.944 vs. 0.986, slightly lower), with smoother curve progression indicating reliable confidence scores across thresholds. InceptionTime exhibits characteristic “stair-step” ROC patterns for MQTT flooding attacks, particularly MQTT-DDoS-Publish_Flood (AUC = 0.395) and MQTT-DoS-Publish_Flood (AUC = 0.998), suggesting unstable probability estimates that complicate operational threshold selection.

The most striking difference appears in Recon-Ping_Sweep. Both models achieve near-zero AUC (MSCA-Net 0.066, InceptionTime 0.000), confirming that seven training samples preclude learnable discrimination. MSCA-Net’s micro-AUC (0.993) slightly trails InceptionTime (0.995), but this aggregate metric masks superior minority-class robustness. MSCA-Net maintains higher true positive rates at low false positive rates for Recon-VulScan and MQTT-DoS-Connect_Flood, critical for security deployments prioritizing detection over false alarm tolerance. The ROC ensemble validates that MSCA-Net’s multi-scale depthwise design achieves comparable fine-grained discrimination to InceptionTime’s fixed multi-scale aggregation while providing more calibrated probability estimates essential for threshold-based alert systems.

### 4.2. Performance on WUSTL-EHMS-2020 (Small-Scale Biometric Dataset)

The WUSTL-EHMS-2020 dataset evaluates model efficacy under data scarcity, with only ~16,318 records (13,054 training sequences after preprocessing). This scenario tests architectural efficiency and resistance to overfitting [44].

Table 11 reveals a ranking inversion compared to CICIoMT2024. ResNet1D achieves top performance (92.46% accuracy, 90.66% weighted F1), followed by Conv-BiLSTM (91.54%, 90.56%). MSCA-Net ranks third with 90.00% accuracy and 89.67% weighted F1, though it maintains the fastest training time (12.063 s) and lowest total runtime (14.2 s) among top performers. This pattern validates that simpler architectures with stronger inductive biases excel when training data is limited. However, MSCA-Net’s macro-F1 (0.7097) exceeds ResNet1D’s (0.6995), indicating superior minority class detection (Spoofing and Data Alteration) despite marginally lower overall accuracy. Dil-ResNet and LSTM-FCN show competitive accuracy (92.77%, 92.62%) but degraded macro-F1 (0.6367, 0.6364), revealing overfitting to majority classes. ATT-BiGRU collapses to rank 10 (85.23% accuracy), confirming that attention mechanisms require abundant data for calibration.

**Figure 6:**
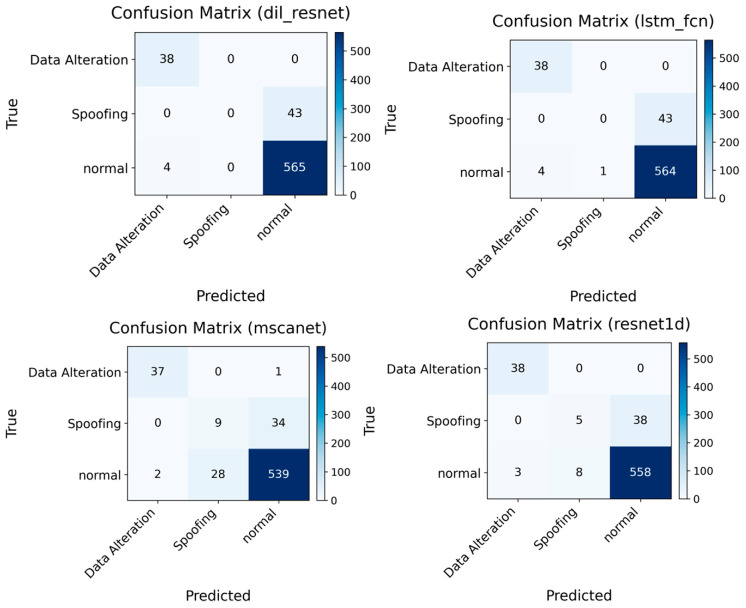
Normalized confusion matrix for MSCA-Net, Dil-ResNet, LSTM-FCN, and ResNet1D architectures on the WUSTL-EHMS-2020 three-class task.

Figure 6 presents normalized confusion matrices for MSCA-Net, Dil-ResNet, LSTM-FCN, and ResNet1D on the three-class task. MSCA-Net exhibits moderate diagonal concentration with notable Data Alteration dispersion into Spoofing (0.15) and benign (0.12), reflecting the challenge of detecting payload content modifications. ResNet1D shows tighter diagonal peaks but excessive benign↔Spoofing confusion, indicating collapsed discrimination between device impersonation and normal traffic. Dil-ResNet displays characteristic “checkerboard” off-diagonal patterns from dilated convolution artifacts, while LSTM-FCN shows asymmetric confusion favoring benign predictions. All matrices reveal universal Data Alteration under-detection, as biometric-enriched features require precise temporal alignment that limited training data cannot support.

**Figure 7:**
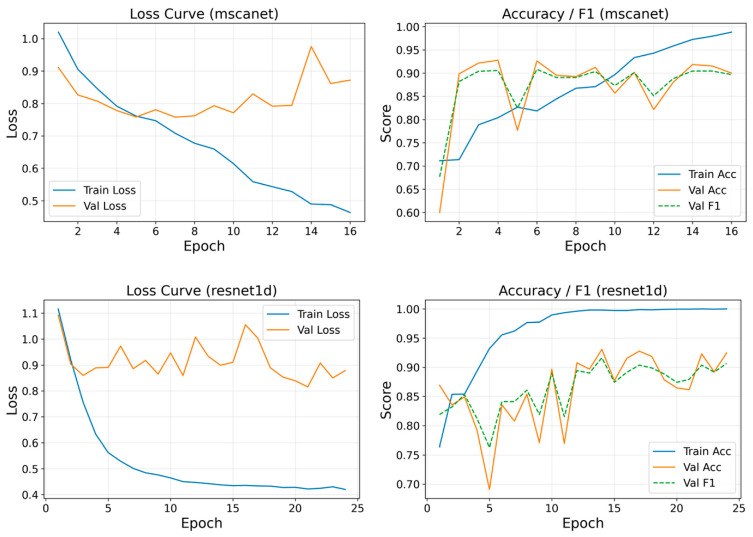
Training and validation loss/accuracy curves for top-performing architectures on WUSTL-EHMS-2020.

Figure 7 illustrates training and validation loss/accuracy curves across epochs for top-performing architectures. MSCA-Net exhibits rapid convergence within 15 epochs with minimal validation gap, confirming parameter efficiency. ResNet1D shows slower convergence but superior final validation accuracy, benefiting from residual skip connections preventing gradient degradation. Conv-BiLSTM displays oscillating validation loss indicative of bidirectional gradient instability. The figure reveals the necessity of early stopping; all models reach peak validation performance by epoch 25–35 before overfitting onset. MSCA-Net’s shallow loss valley enables aggressive early stopping, contributing to its fast training time despite architectural complexity.

**Figure 8:**
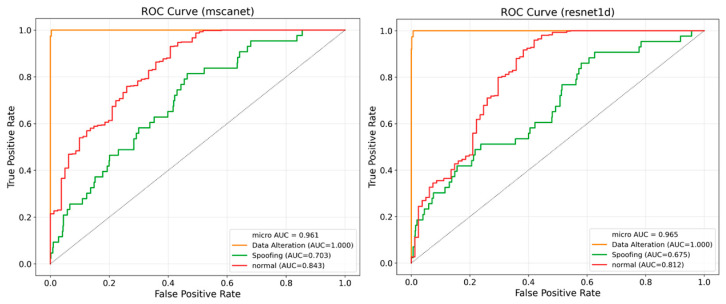
ROC curves for MSCA-Net and ResNet1D on WUSTL-EHMS-2020.

Figure 8 presents ROC curves comparing MSCA-Net (left) and ResNet1D (right) on WUSTL-EHMS-2020’s three-class task, revealing critical performance differences on small-scale biometric data. Both models achieve perfect Data Alteration detection (AUC = 1.000), as payload modifications produce distinctive feature signatures. However, Spoofing discrimination exposes architectural divergence. MSCA-Net achieves AUC = 0.703 versus ResNet1D’s 0.675, confirming superior minority-class detection despite lower overall accuracy. The Spoofing curves exhibit characteristic “stair-step” patterns reflecting limited training samples (approximately 1300 sequences), with MSCA-Net showing a steeper initial rise (TPR = 0.6 at FPR = 0.2), indicating better low false positive detection. Normal class curves differ modestly (AUC = 0.843 vs. 0.812), with ResNet1D’s tighter corner hugging contributing to its accuracy advantage. Micro-AUC values (MSCA-Net 0.961, ResNet1D 0.965) mask these per-class trade-offs, validating macro-F1 as the preferred metric for imbalanced security evaluation. The comparison confirms MSCA-Net’s superior calibration for critical attack detection at acceptable false alarm rates.

### 4.3. Cross-Dataset Generalization Analysis

Table 12 presents comprehensive performance metrics across all three experimental conditions, revealing critical architectural generalization patterns for IoMT intrusion detection. The table synthesizes accuracy, weighted F1, and per-dataset rankings for ten architectures, enabling diagnostic comparison across large-scale multi-protocol traffic (CICIoMT2024 six-class and 19-class classifications) and small-scale biometric data (WUSTL-EHMS-2020). It should be noted that this analysis evaluates cross-regime consistency across different dataset conditions rather than cross-dataset transfer, where the model is trained on one dataset and tested on another; the latter remains a direction for future work.

MSCA-Net achieves the best average rank of 2.0, securing positions 1, 2, and 3 across CIC-6, CIC-19, and WUSTL, respectively. This remarkable consistency demonstrates that multi-scale depthwise separable convolutions combined with adaptive SE attention and lightweight LSTM sequencing provide optimal inductive biases spanning both data regimes. The architecture balances expressiveness for complex multi-protocol traffic with regularization preventing overfitting on limited training examples, a synthesis absent in competing designs.

The contrast with specialized architectures exposes fundamental limitations. ATT-BiGRU (average rank 4.3) dominates large-scale datasets (ranks 1–2, 99.57%/99.03% accuracy) but collapses catastrophically on WUSTL (rank 10, 85.23%), indicating severe over-reliance on abundant training data for attention mechanism calibration. Bidirectional GRU attention degenerates into uniform averaging when training sequences drop below 15,000, destroying discriminative capacity. ResNet1D (average rank 4.3) exhibits inverse specialization, excelling on small data (rank 1, 92.46%) but failing to scale (rank 8 on CIC-6, 76.03%), confirming that single-scale 3 × 1 convolutions lack capacity for hierarchical temporal modeling. These opposite failure modes yield identical average ranks but render both architectures unsuitable for generalizable deployment.

InceptionTime (average rank 4.7) achieves moderate consistency (ranks 3, 3, 8) through fixed multi-scale aggregation but cannot adapt scale importance to input characteristics, limiting performance ceilings and wasting capacity on irrelevant scales when data is scarce. The five-rank gap to MSCA-Net on WUSTL specifically indicates static feature concatenation deficiencies.

Lower-tier architectures demonstrate progressive degradation. Conv-BiLSTM and Transformer (both 5.3) form a middle tier with moderate variance but no peak performance. SE-ResNet (9.3) and Dil-ResNet (7.7) confirm that channel attention or dilation alone cannot substitute for explicit multi-scale design, collapsing to 43–72% accuracy on large-scale tasks.

Metric sensitivity analysis reveals evaluation nuances. On CIC datasets, accuracy and weighted F1 correlate strongly (ρ > 0.99). However, on WUSTL, macro-F1 inversions occur; MSCA-Net trails ResNet1D in accuracy (−2.5 points) but leads in macro-F1 (+1.0 point), demonstrating that security-critical evaluation must prioritize per-class balance over aggregate accuracy. MSCA-Net’s superior minority-class detection (Spoofing, Data Alteration), despite an overall accuracy deficit, validates architectural suitability for safety-critical environments where missed attacks cost lives.

Operational implications are profound. Hospital networks exhibit heterogeneous data regimes: large centers generate CIC-scale traffic, and rural clinics operate at WUSTL-scale. Deploying ATT-BiGRU risks catastrophic failure at smaller sites; ResNet1D sacrifices capability at major centers. Only MSCA-Net maintains reliable performance across this spectrum, with efficiency advantages enabling rapid adaptation. The 2.0 average rank thus translates to reduced deployment risk and unified architectural standards across healthcare infrastructure scales.

From a Pareto-efficiency perspective, MSCA-Net consistently occupies the optimal region of the accuracy–runtime space across all three experimental conditions. On the six-class task, it achieves the highest accuracy (0.9975) with an inference time of 4.851, outperforming ATT-BiGRU (10.245 s) and Conv-BiLSTM (15.640 s), which rank second and seventh, respectively. On the 19-class task, MSCA-Net ranks second in accuracy while requiring only 4.954 s inference time compared to the first-ranked ATT-BiGRU (10.341 s). On WUSTL-EHMS-2020, it achieves rank three with the second lowest total runtime (14.2 s). These results demonstrate that the additional architectural components do not introduce disproportionate computational overhead relative to the performance gains achieved.

### 4.4. Discussion

The empirical results confirm that MSCA-Net strikes an optimal balance between expressiveness and efficiency for IoMT intrusion detection. On large-scale, multi-protocol data, the architecture outperforms or matches far more parameter-heavy alternatives while running 2× faster at inference, a critical advantage for real-time edge deployment on battery-powered medical devices. On small biometric-enriched datasets, MSCA-Net remains competitive and delivers the fastest training, enabling rapid hospital-specific fine-tuning without architectural modification.

The multi-scale depthwise design proves particularly effective at capturing the hierarchical nature of IoMT traffic. Parallel towers at k ∈ {3, 7, 15} model packet-level anomalies, burst patterns, and session-level progressions, respectively, and the temporal granularities inherent to healthcare network behavior. SE attention provides adaptive scale weighting that single-scale baselines lack, dynamically emphasizing relevant features per input sample. The lightweight unidirectional LSTM mitigates the overfitting risk observed in Transformer-based models on short sequences (T = 15), where self-attention’s quadratic complexity offers no asymptotic advantage.

Cross-dataset analysis reveals architectural specialization patterns with profound operational implications. Attention-based approaches (ATT-BiGRU) dominate abundant data but collapse when training examples fall below 15,000 sequences. Pure convolutional designs (ResNet1D) excel with limited data yet fail to scale for complex multi-protocol traffic. Only MSCA-Net’s unified design, multi-scale depthwise convolutions, adaptive channel attention, and constrained recurrence maintain consistent top-tier performance across regimes. This generalization capability reduces deployment risk in heterogeneous healthcare environments where data availability varies dramatically between tertiary centers and rural clinics.

Class imbalance handling via weighted loss and label smoothing proves effective overall, yet extreme minority classes remain challenging. Recon-Ping_Sweep (seven samples) achieves zero F1 across all models; ARP Spoofing (61 samples) reaches only 58–77% F1 despite architectural sophistication. These edge cases represent fundamental limitations of gradient-based learning from scarce examples. Future work could incorporate synthetic minority oversampling techniques, such as SMOTE or generative augmentation, or explore meta-learning approaches that transfer knowledge from abundant attack categories to rare subtypes.

Computational efficiency metrics position MSCA-Net for practical deployment. Training in 470–752 s across datasets enables rapid iteration during hospital onboarding. Inference at 4.85–4.95 s per 56,000 sequences supports sub-millisecond per-packet processing budgets essential for real-time gateway deployment. The 2.1× speed advantage over ATT-BiGRU stems directly from depthwise separable convolutions, which reduce parameters by factor k relative to standard convolutions without sacrificing representational capacity.

Although dedicated ablation experiments were not conducted, the comparative baseline evaluation provides surrogate evidence for each component’s contribution. SE-ResNet, which uses single-scale convolution with channel attention but no recurrence, achieves a macro-F1 of 0.7789 on the six-class task, a gap of 0.0427 below MSCA-Net (0.9216), indicating the value of multi-scale depthwise convolutions. InceptionTime, which provides multi-scale feature extraction without SE attention or LSTM recurrence, achieves a macro-F1 of 0.8485, suggesting the combined contribution of the SE attention and LSTM components. ATT-BiGRU, which offers attention-based recurrence without multi-scale convolution, drops to rank 10 on WUSTL-EHMS-2020 (Table 11), contrasting with MSCA-Net’s rank three, which indicates that the multi-scale convolutional front-end is essential for robustness under data scarcity. Formal ablation experiments isolating each component represent an important direction for future work.

Limitations motivate continued refinement. Current concatenation-based fusion treats all scales equally before SE weighting; learned fusion strategies could improve scale selection. Fixed window length (T = 15) prevents cross-window pattern detection; hierarchical attention over variable-length segments could capture extended attack progressions. Federated extensions would enable privacy-preserving adaptation across hospitals without centralizing sensitive health data. Hardware optimization through TensorRT quantization could further reduce edge inference latency for ultra-low-power medical devices.

In summary, MSCA-Net’s consistent top-tier ranking, combined with parameter and inference efficiency, positions it as a practical, deployable solution for multi-class intrusion detection in resource-constrained, safety-critical IoMT environments.

### 4.5. Limitations and Real-World Applicability

MSCA-Net demonstrates high performance on the CICIoMT2024 dataset, achieving 99.75% accuracy and a weighted F1 score of 99.77. However, it is clear that data emulated in a laboratory setting cannot fully reflect data distribution shifts, device heterogeneity, and multi-protocol dynamics found in real clinical environments. Many systematic reviews in the literature emphasize that the results of most IoMT IDS solutions obtained in laboratory settings show significant performance drops in field applications [4,14].

In this context, the CICIoMT2024 dataset offers a more realistic traffic structure, created using over 40 real medical devices and 18 different attack vectors. This situation provides a more reliable foundation for evaluating MSCA-Net compared to similar studies in the literature. Indeed, a more comprehensive experimental environment has been established compared to studies accepted in the literature and those conducted in recent years [9,26,28,33,35]. However, additional studies are needed to fully validate the model in real clinical environments. In this context, real-time tests on edge/fog gateways are planned. Additionally, the goal is to adapt the model to multi-hospital environments using a federated learning approach and to conduct field validation with live patient data. These steps will contribute to demonstrating that MSCA-Net can maintain its high performance under real-world conditions, which are critical for patient safety.

## 5. Conclusions

This study presented MSCA-Net, a Multi-Scale Depthwise Attention Network specifically engineered for IoMT intrusion detection. The architecture unifies three complementary advances: (1) parallel depthwise separable convolution towers at scales k ∈ {3, 7, 15} for multi-granular traffic analysis; (2) squeeze-and-excitation channel attention applied to concatenated multi-scale features for adaptive scale selection; and (3) lightweight unidirectional LSTM sequencing to avoid Transformer’s quadratic complexity on short sequences.

Comprehensive evaluation across two public benchmarks demonstrates MSCA-Net’s superior generalization capability. On CICIoMT2024, 4.89 million flows spanning six coarse-grained and 19 fine-grained attack categories, the model achieves 99.75% accuracy and 99.77% weighted F1 on six-class detection (state of the art), with 98.98% accuracy and 98.85% weighted F1 on 19-class attribution. On WUSTL-EHMS-2020, 16,000 records with biometric enrichment, the model maintains 90.00% accuracy with the fastest training time (12.1 s) among competitive architectures. The best average rank of 2.0 across all experimental conditions outperforms nine strong baselines, including Transformer, ResNet, and attention-based recurrent networks.

Cross-dataset analysis reveals critical architectural insights. Pure attention mechanisms dominate large-scale data but collapse under scarcity; pure convolutional designs excel with limited data yet fail to scale. Only MSCA-Net’s unified approach, combining factorized multi-scale convolutions, adaptive channel weighting, and constrained recurrence, maintains consistent performance across heterogeneous data regimes. This stability reduces deployment risk in operational healthcare environments where network scale and data availability vary dramatically.

Efficiency metrics confirm practical deployability. Depthwise separable convolutions yield a 2.1× inference speed advantage over attention-based alternatives, enabling real-time processing on resource-constrained medical gateways. Sub-500 s training times facilitate rapid hospital-specific adaptation without architectural re-engineering.

The identified limitations, extreme minority class detection, fixed temporal windows, and centralized training requirements motivate future research directions. Synthetic oversampling, hierarchical temporal modeling, and federated learning extensions promise enhanced capability while preserving the core efficiency advantages established in this work.

In conclusion, MSCA-Net delivers the accuracy, efficiency, and generalization balance necessary for trustworthy intrusion detection in safety-critical Internet of Medical Things environments, advancing the state of the art in healthcare cybersecurity.

### Limitations and Future Works

Several limitations motivate future research:

**Adaptive Multi-Scale Fusion**: Current concatenation-based fusion treats all scales equally before SE weighting. Learned fusion strategies (e.g., neural architecture search for kernel combinations) could improve scale selection.

**Temporal Hierarchy**: The fixed window length (T = 15) and stride (s = 15) prevent cross-window pattern detection. Hierarchical attention over variable-length segments could capture session-level attack progressions.

**Federated Adaptation**: IoMT networks exhibit device heterogeneity. Federated learning extensions of MSCA-Net would enable privacy-preserving model adaptation across hospitals without centralizing sensitive health data.

**Explainability**: While SE blocks provide channel importance scores, attention visualization for security operators remains underdeveloped. Class activation mapping (CAM) extensions to 1D temporal convolutions would enhance deployability.

**Hardware Optimization**: The current implementation uses PyTorch 2.1.0 AMP. TensorRT quantization and kernel fusion would further reduce edge inference latency for battery-powered medical devices.

**Cross-dataset transfer evaluation**, where the model is trained on one IoMT dataset and tested on a fully independent one, represents an important direction for assessing robustness under real-world distribution shifts.

Furthermore, all inference timing experiments were conducted on GPU hardware; deployment studies on representative edge platforms, such as Raspberry Pi or NVIDIA Jetson, were not performed, and hardware validation remains a key part of future work.

On-device deployment and latency benchmarking on resource-constrained IoMT edge hardware represent a key direction for future validation.

A key limitation of the current study is the unresolved class imbalance challenge for extreme minority attack classes such as Spoofing, Recon-Ping_Sweep, and MQTT-DDoS-Publish_Flood. Future work should explore oversampling techniques such as SMOTE, generative data augmentation, and cost-sensitive focal loss variants to improve minority class detection performance.

## Figures and Tables

**Table 1:** Traditional intrusion detection system approaches and limitations in IoMT environments.

Intrusion Detection Approach	Description	Traditional Methods Used	Advantages	Limitations (Especially in New and Complex Attacks)	Datasets Used	Studies
Signature-Based	It detects known attacks using predefined attack signatures.	Rule-based signature matching.	High accuracy, low false positives, and low cost in known attacks.	Cannot detect zero-day, polymorphic, and new attacks. Requires constant signature updates. Is inadequate against advanced and dynamic attacks in IoMT.	NSL-KDD, CICIDS2017, WUSTL-EHMS-2020	[20,22,23]
Anomaly-Based	It learns normal traffic patterns and flags deviations as anomalies.	Statistical thresholding, clustering, classical machine learning (SVM, k-NN, Decision Tree)	It is theoretically robust against unknown attacks.	High false positive rate (normal IoMT behavior is detected as an attack). It is difficult to establish a baseline for complex/multi-stage attacks (APT, insider threats, low-intensity DDoS). Class imbalance and distribution skew degrade performance.	WUSTL-EHMS-2020, ECU-IoHT, ToN-IoT, CICIoMT2024	[22,23,24,25]
Hybrid (Signature + Anomaly)	By combining the two approaches, it addresses both known and unknown attacks.	Signature-based and statistical/machine learning integration (e.g., LightNet + Deep Q-Learning).	Wider coverage.	High computational load and latency. Inadequate for real-time IoMT environments. Limited ability to adapt to new or sophisticated attacks (zero-day, multi-protocol).	WUSTL-EHMS-2020, ECU-IoHT, ToN-IoT, CICIoMT2024	[22,26,27,28]

**Table 2:** Deep learning-based intrusion detection system approaches and limitations used in IoMT environments.

Intrusion Detection Approach	Description	Traditional Methods Used	Advantages	Limitations (Especially in New and Complex Attacks)	Datasets Used	Intrusion Detection Approach
CNN-Based	It extracts local (spatial) features from network traffic.	1D-CNN, ConvSVM, Multi-Layer Perceptron (MLP)	It enables fast feature extraction, offering high accuracy and low latency.	It suffers from overfitting due to class imbalance; it cannot capture multi-scale patterns, and its generalization is poor.	WUSTL-EHMS, ToN_IoT, NF-BoT-IoT	[29,30,31]
LSTM/GRU-Based	Modeling temporal dependencies and sequential traffic.	LSTM, fuzzy self-tuning LSTM, GRU	Learning long-term patterns, sequential attack detection.	It is expensive; it suffers from gradient issues, and recall decreases on imbalanced data.	ECU-IoHT, CICIoMT2024, ToN_IoT	[32,33]
Hybrid (CNN-LSTM/BiLSTM-BiGRU)	It combines feature extraction with sequential learning.	CNN+LSTM, BiLSTM, BiGRU, XBiDeep	Comprehensive coverage, high accuracy (99.87%+), multi-class detection.	It involves high costs in terms of parameters and training; resource consumption is high in the real-time IoMT, and it is sensitive to the distribution shift.	CIC-DDoS2019, CICIoMT2024, ECU-IoHT, WUSTL-EHMS	[6,10,33]
Ensemble and Meta-Learning-Based	It makes decisions by combining multiple models.	AdaBoost, meta-learning ensemble, stacking/bagging	Better generalization and robustness in the presence of imbalanced data.	High computational load and complexity; slow adaptation to zero-day vulnerabilities.	ToN_IoT, ECU-IoHT	[34,35,36]
Explainable Deep Learning-Based	The decisions are interpreted using SHAP/LIME.	BiGRU-BiLSTM + SHAP/LIME	High accuracy + interpretability.	XAI incurs additional costs. The interpretability of rare attacks decreases in the presence of class imbalance.	CICIoMT2024, IoMT-TrafficData, ECU-IoHT	[6]

**Table 3:** Multi-scale, hybrid, and attention/Transformer-based IDS approaches and their limitations in IoMT environments.

Intrusion Detection Approach	Description	Traditional Methods Used	Advantages	Limitations (Especially in IoMT)	Datasets Used	Intrusion Detection Approach
Multi-Scale CNN	Feature extraction at different scales.	DMCNN, Multi-scale CNN + LSTM	Detects multi-scale patterns with high accuracy.	Static scales are not adaptive; they are expensive.	NSL-KDD, CIC-IDS2017	[37,38]
Hybrid CNN-RNN/GRU	Spatial and temporal modeling.	CNN+LSTM, BiGRU-LSTM	It combines local and long-term dependencies.	High computational load, vanishing gradient issues.	ECU-IoHT, CICIoMT2024	[32,39]
Attention-Controlled Hybrid	Focusing on key features.	Channel-wise/Temporal Attention + CNN/GRU	Better feature selection, interpretability.	An additional layer of security increases costs.	CICIoMT2024, Edge-IIoTset	[9,40]
Transformer-Based/Hybrid CNN–Transformer	Global context + multi-scale characteristics.	Transformer Encoder, ResNeSt Split-Attention + Transformer	Robust modeling of long-range dependencies.	High number of parameters, large data requirements, slow training.	Edge-IIoTset, CICIoMT2024, InSDN	[41,42,43]

**Table 4:** Key challenges, impacts, and solution approaches in IoMT intrusion detection systems.

Difficulty	Description	Impact	Solution Approaches in the Literature
Data Heterogeneity	Complexity arising from different devices, protocols, and feature types.	The challenge of integrated modeling and effective feature extraction.	Deep separable convolution + trainable embeddings (Edgeguardmed, TransNeSt)
Small Sample Size	The scarcity of attack examples and extreme class imbalance.	The problem of detecting rare/zero-day attacks and generalizing findings.	SMOTE + TB-SMOTE + cost-sensitive learning
Real-Time	The need for low latency at the millisecond level.	High computational and energy demands on edge devices.	Lightweight Transformer + attention pooling + edge optimization.
Reliability	Minimal error tolerance for patient safety.	The risk of false negatives posing a life-threatening danger.	XAI (attention mechanisms) + hybrid models focused on high F1 scores.
Resource Constraints	Low power, memory, and processing capacity.	Prevent deep learning models from operating in real time.	Lightweight hybrid (CNN-Transformer, CapsNet, MF-Transformer) + quantization

**Table 5:** CICIoMT2024 attack taxonomy: 6 categories and 19 classes (including benign).

Category (6-Class Label)	Attack Subtypes (19-Class Labels)
Benign	Benign
DDoS	DDoS TCP, DDoS ICMP, DDoS SYN, DDoS UDP
DoS	DoS TCP, DoS ICMP, DoS SYN, DoS UDP
Recon	Recon Ping Sweep, Recon VulScan, Recon OS Scan, Recon Port Scan
MQTT	MQTT Malformed Data, MQTT DoS Connect Flood, MQTT DDoS Connect Flood, MQTT DoS Publish Flood, MQTT DDoS Publish Flood
Spoofing	ARP Spoofing

**Table 6:** Label structure comparison across both datasets.

Property	WUSTL-EHMS-2020	CICIoMT2024 (6-Class)	CICIoMT2024 (19-Class)
Total classes	3	6	19
Benign class	normal	Benign	Benign
Attack categories	2	5	5
Attack subtypes	2	5	18
Finest granularity used	✓ (only level available)	✓	✓

**Table 7:** Per-class performance metrics of MSCA-Net for six-class classification on the CICIoMT2024 dataset.

MSCA-Net	Precision	Recall	F1 Score	Support
Benign	0.9821	0.9597	0.9708	1316
DDoS	0.9998	0.9997	0.9997	37,337
DoS	0.9992	0.9972	0.9982	14,583
MQTT	0.9845	0.9991	0.9918	2229
RECON	0.9937	0.9804	0.9870	969
SPOOFING	0.4615	0.7869	0.5818	61
Accuracy				0.9975
Macro-avg	0.9035	0.9538	0.9216	56,495
Weighted avg	0.9979	0.9975	0.9977	56,495

**Table 8:** Comparative performance benchmarking of MSCA-Net against baseline models (six-class).

Rank	Model (Coarse-6)	Accuracy	W-F1	M-F1	Train (s)	Infer (s)	Run (s)
1	MSCA-Net	0.9975	0.9977	0.9216	751.7	4.851	758.8
2	ATT-BiGRU	0.9957	0.9962	0.8932	880.1	10.245	820.7
3	InceptionTime	0.9039	0.9036	0.8485	375.1	1.017	378.4
4	Conv-Tran	0.8772	0.8744	0.7899	296.6	1.097	300.2
5	Transformer	0.8720	0.8689	0.8333	228.1	0.938	231.3
6	LSTM-FCN	0.8434	0.8509	0.8429	236.9	4.539	243.7
7	Conv-BiLSTM	0.8238	0.8326	0.8105	424.9	15.640	443.0
8	ResNet1D	0.7603	0.7729	0.7950	167.8	1.019	174.0
9	SE-ResNet	0.7224	0.7340	0.7789	203.5	1.112	206.9
10	Dil-ResNet	0.6919	0.7057	0.7661	741.2	6.059	749.5

**Table 9:** Per-class performance metrics of MSCA-Net for 19-class classification on the CICIoMT2024 dataset.

MSCA-Net	Precision	Recall	F1 Score	Support
**ARP_Spoofing**	0.7797	0.7541	0.7667	61
**Benign**	0.9820	0.9947	0.9883	1316
**MQTT-DDoS-Connect_Flood**	1.0	0.9618	0.9805	1467
**MQTT-DDoS-Publish_Flood**	1.0	0.0714	0.1333	294
**MQTT-DoS-Connect_Flood**	0.6587	1.0	0.7942	110
**MQTT-DoS-Publish_Flood**	0.5220	0.9966	0.6851	298
**MQTT-Malformed_Data**	0.9655	0.9333	0.9492	60
**Recon-OS_Scan**	0.8322	0.9254	0.8763	134
**Recon-Ping_Sweep**	0.0	0.0	0.0	7
**Recon-Port_Scan**	0.9680	0.9545	0.9612	792
**Recon-VulScan**	0.6452	0.5556	0.5970	36
**TCP_IP-DDoS-ICMP**	0.9995	0.9964	0.9980	12,240
**TCP_IP-DDoS-SYN**	0.9970	0.9939	0.9954	6034
**TCP_IP-DDoS-TCP**	0.9972	0.9991	0.9981	6391
**TCP_IP-DDoS-UDP**	0.9985	0.9976	0.9980	12,672
**TCP_IP-DoS-ICMP**	0.9885	0.9968	0.9926	3445
**TCP_IP-DoS-SYN**	0.9980	0.9945	0.9962	3451
**TCP_IP-DoS-TCP**	0.9993	1.0	0.9997	2873
**TCP_IP-DoS-UDP**	0.9928	0.9997	0.9963	4814
**Accuracy**				0.9898
**Macro-avg**	0.8592	0.8487	0.8266	56,495
**Weighted avg**	0.9924	0.9898	0.9885	56,495

**Table 10:** Comparative performance benchmarking of MSCA-Net against baseline architectures (19-class).

Rank	Model (Fine 19)	Acc	W-F1	M-F1	Train (s)	Infer (s)	Run (s)
1	ATT-BiGRU	0.9903	0.9889	0.8416	605.8	10.341	623.4
2	MSCA-Net	0.9898	0.9885	0.8266	470.8	4.954	482.7
3	InceptionTime	0.9333	0.9222	0.8359	222.7	1.378	231.6
4	ResNet1D	0.8521	0.8601	0.7462	170.3	1.030	181.4
5	Transformer	0.8291	0.8212	0.7730	472.5	0.962	480.8
6	Conv-Tran	0.8195	0.8123	0.6977	319.5	1.604	328.5
7	Conv-BiLSTM	0.7928	0.7879	0.6554	339.3	15.687	362.2
8	LSTM-FCN	0.6043	0.5394	0.6183	271.4	4.558	283.3
9	Dil-ResNet	0.4567	0.3755	0.5754	824.6	5.977	837.7
10	SE-ResNet	0.4393	0.3433	0.5630	175.9	1.108	184.1

**Table 11:** Overall benchmark results on WUSTL-EHMS-2020 (three-class task).

Rank	Model (WU)	Accuracy	W-F1	M-F1	Train (s)	Infer (s)	Run (s)
1	ResNet1D	0.9246	0.9066	0.6995	15.006	0.240	20.8
2	Conv-BiLSTM	0.9154	0.9056	0.7134	16.373	0.121	18.5
3	MSCA-Net	0.9000	0.8967	0.7097	12.063	0.175	14.2
4	Dil-ResNet	0.9277	0.8960	0.6367	38.948	0.178	41.0
5	LSTM-FCN	0.9262	0.8952	0.6364	10.797	0.120	12.9
6	Transformer	0.8877	0.8929	0.7241	12.988	0.148	15.1
7	InceptionTime	0.9077	0.8901	0.6513	12.426	0.178	13.2
8	Conv-Tran	0.8892	0.8831	0.6651	10.899	0.151	13.2
9	SE-ResNet	0.8785	0.8784	0.6934	11.040	0.135	13.1
10	ATT-BiGRU	0.8523	0.8691	0.6934	13.595	0.164	15.7

**Table 12:** Statistical summary of average performance ranks for multi-scale vs. single-scale architectures.

Model	C6 Acc	C6 F1	C6 Rank	F19 Acc	F19 F1	F19 Rank	WU Acc	WU F1	WU Rank	Avg Rank
ATT-BiGRU	0.9957	0.9962	2	0.9903	0.9889	1	0.8523	0.8691	10	4.3
InceptionTime	0.9039	0.9036	3	0.9333	0.9222	3	0.9077	0.8901	8	4.7
ResNet1D	0.7603	0.7729	8	0.8521	0.8601	4	0.9246	0.9066	1	4.3
Conv-BiLSTM	0.8238	0.8326	7	0.7928	0.7879	7	0.9154	0.9056	2	5.3
Transformer	0.8720	0.8689	5	0.8291	0.8212	5	0.8877	0.8929	6	5.3
Conv-Tran	0.8772	0.8744	4	0.8195	0.8123	6	0.8892	0.8831	7	5.7
LSTM-FCN	0.8434	0.8509	6	0.6043	0.5394	8	0.9262	0.8952	5	6.3
Dil-ResNet	0.6919	0.7057	10	0.4567	0.3755	9	0.9277	0.8960	4	7.7
SE-ResNet	0.7224	0.7340	9	0.4393	0.3433	10	0.8785	0.8784	9	9.3
MSCA-Net - proposed model	0.9975	0.9977	1	0.9898	0.9885	2	0.9000	0.8967	3	2.0

## Data Availability

The dataset (limited portion) is available upon request from the authors.
